# A Prosthetic Hand Body Area Controller Based on Efficient Pattern Recognition Control Strategies

**DOI:** 10.3390/s17040869

**Published:** 2017-04-15

**Authors:** Simone Benatti, Bojan Milosevic, Elisabetta Farella, Emanuele Gruppioni, Luca Benini

**Affiliations:** 1 Micrel Lab, Unversity of Bologna, 40126 Bologna, Italy; luca.benini@unibo.it; 2 E3DA, Fondazione Bruno Kessler, 38123 Trento, Italy; milosevic@fbk.eu(B.M.); efarella@fbk.eu (E.F.); 3 Centro Protesi INAIL, Vigorso di Budrio, 40054 Bologna, Italy; e.gruppioni@inail.it; 4 Integrated Systems Laboratory, ETHZ, 8092 Zurich, Switzerland; lbenini@iis.ee.ethz.ch

**Keywords:** EMG, gesture recognition, prosthetics, BSN, human machine interaction

## Abstract

Poliarticulated prosthetic hands represent a powerful tool to restore functionality and improve quality of life for upper limb amputees. Such devices offer, on the same wearable node, sensing and actuation capabilities, which are not equally supported by natural interaction and control strategies. The control in state-of-the-art solutions is still performed mainly through complex encoding of gestures in bursts of contractions of the residual forearm muscles, resulting in a non-intuitive Human-Machine Interface (HMI). Recent research efforts explore the use of myoelectric gesture recognition for innovative interaction solutions, however there persists a considerable gap between research evaluation and implementation into successful complete systems. In this paper, we present the design of a wearable prosthetic hand controller, based on intuitive gesture recognition and a custom control strategy. The wearable node directly actuates a poliarticulated hand and wirelessly interacts with a personal gateway (i.e., a smartphone) for the training and personalization of the recognition algorithm. Through the whole system development, we address the challenge of integrating an efficient embedded gesture classifier with a control strategy tailored for an intuitive interaction between the user and the prosthesis. We demonstrate that this combined approach outperforms systems based on mere pattern recognition, since they target the accuracy of a classification algorithm rather than the control of a gesture. The system was fully implemented, tested on healthy and amputee subjects and compared against benchmark repositories. The proposed approach achieves an error rate of 1.6% in the end-to-end real time control of commonly used hand gestures, while complying with the power and performance budget of a low-cost microcontroller.

## 1. Introduction

There are an estimated 2 million hand amputees in the United States and approximately the same in Europe. More than 200,000 new amputation surgeries are performed each year and approximately 10,000 children receive amputations resulting in a lifelong disability [1]. Amputee patients are supported by a long standing research and development of prosthetic devices, which can be divided in passive and active ones. Passive prostheses have only a cosmetic purpose and do not support any of the hand functionalities. Modern active prostheses are externally powered and feature advanced grasping and control functionalities.

The first active prostheses were composed by a split hook able to perform a power grasp, restoring just one Degree of Freedom (DoF). During the last years, the advancement of technology and prosthesis design paved the way for multifinger artificial hands [2,3]. These are poliarticulated systems that independently actuate each finger, reproducing most of the movements of a real hand in daily living activities. They offer high reliability and robustness, but do not provide an intuitive control interface. Electromyography (EMG) is widely used in assistive technologies to sense the muscular activities in the forearm [4] and it is a preferred mode of interaction to control prosthetic devices. However, State-of-the-Art (SoA) systems [2,5,6] use simple thresholds to detect the user’s activity and the prosthesis commands are encoded in predefined sequences of extensions and flexions of the residual forearm muscles [7]. This approach does not offer a natural interface, it requires a long learning curve and high cognitive effort during use.

Furthermore, several studies compare these SoA controllers against natural control strategies, indicating that the design of intuitive controllers can improve the usability and the functionality of a prosthesis [8,9,10,11]. Massive research efforts are spent to investigate solutions for a more intuitive prosthetic control, using techniques based on soft synergies [12] or pattern recognition [13]. Both approaches exploit EMG sensing of the remaining forearm muscles as the main input for the user-prosthesis interface. A prosthetic hand based on synergies offers only one gesture, i.e., an adaptive power grip, however, it provides a dynamic control of the grasping force and the ability to adapt the hand’s grasp to the object in use. On the other hand, the use of pattern recognition and machine learning techniques allows to recognize the intention to perform different gestures, such as pointing and different types of power and precision grips.

Development and optimization of gesture recognition techniques for the control of poliarticulated prostheses is today an active research topic, even though the analysis is mainly performed from a machine learning perspective [14,15,16]. Such studies focus on the offline analysis and evaluation of the algorithm’s recognition accuracy on all the samples of a given dataset. However, this is not sufficient for the exhaustive evaluation of a complete prosthetic controller, which requires a system approach involving sensing, classification and timely prosthesis actuation on a wearable system for a correct execution of gestures.

Even if integration advancement enables single wearable devices to have unprecedented computational power, the case of EMG gesture recognition still presents a processing power challenge to these devices, in particular in the training phase of the classifier and therefore requires to be supported by a gateway device.

In this paper, we present the design of a Body Sensor Network (BSN) for the control of a poliarticulated prosthetic hand. This work is the final result of a two year collaboration project between two research partners (University of Bologna and Fondazione Bruno Kessler) and the INAIL prosthetic center, a leading European institute for prosthetics and rehabilitation. The work covers the whole system development, starting from the EMG data collection on healthy and amputee subjects [17] and going through the study and the embedded implementation of an efficient pattern recognition algorithm. We focus on the design of a natural method of interaction between amputees and prosthesis, which is integrated with a robust real-time control strategy and actuation of a multifinger hand. The wearable node is part of the BSN and it is connected via Bluetooth to a personal gateway (e.g., a smartphone) for the personalized training and tuning of the recognition capabilities. The gateway is a second node that can also open the BSN to context-aware information and make the BSN cloud assisted [18,19,20]. Furthermore, the gateway node enables periodic classifier retraining in daily life conditions, which is a process that does not require real-time as the recognition in the wearable node. Such approach is in line with the BSNs and wearables trend of embedding processing near the sensor and combining with cloud technologies to enable big data analysis for the IoT scenarios [21,22,23,24,25].

The performance of the system has been tested in terms of *end-to-end* recognition ratio, evaluating the accuracy and timing of the system to recognize and execute complete hand gestures, as performed by amputees and healthy subjects. The system classifies and executes four hand gestures (open hand, closed hand, precision grasp and point index) with an end-to-end error rate under 2%. It has been tested on five healthy subjects and four amputee patients. Moreover, we validated our approach against the NINAPRO database that represents the largest and most complete dataset for EMG gesture recognition to date [26]. The proposed system is designed with Commercial Of The Shelf (COTS) components and open source development tools, to make it easy to replicate all the results and to provide an useful platform for future research and product development.

The reminder of this paper is organized as follows: Section 2 contains background information and related work, Section 3 reports a detailed description of the proposed system and approach and Section 4 presents its experimental evaluation. Finally, in Section 5 we draw the conclusions.

## 2. Background and Related Work

The hand prosthesis acts as functional replacement of the missing limb in amputees. Technological advancements over the years allowed significant improvements in the restoration of the functionalities of a normal human hand. The first example of these active devices are the body-powered prostheses, capable of restoring basic tasks such as opening and closing a terminal device [27]. In such devices, motion is transmitted to the prosthesis mechanically, controlling the artificial hand with the abduction of the shoulder or the healthy wrist flexion. These devices are often used because they are simple, robust, and relatively inexpensive, even though they do not re-establish a complete functionality of the hand.

Electrically powered or active prostheses [28] are advantageous w.r.t. body-powered ones because they require less user effort, as movement is actuated with DC motors [29]. They can be controlled through a variety of means such as force sensors [30], acoustic interfaces [31] and EMG signals [32]. Such devices restore some functionality to amputees, but their control is typically limited to only one or two DoFs. Hence, the research on upper limb prostheses addresses multiple challenges in the development of anthropomorphic prostheses, ranging from the hand design [33] to the fingers’ actuation [34], in search of the best tradeoff between the complexity of the design and its usability and reliability.

Recently, the biomechatronic design of these prostheses has been refined, and advanced and reliable multifinger prostheses appeared on the market. The Bebionic 3 [6], the Michelangelo Hand [5] and the I-limb [2] are poliarticulated myoelectric hands with embedded control, which feature from 7 to 24 different hand positions. The fingers are independently driven by dedicated DC motors and, exploiting their robust design, these hands can carry up to 45 kg. A dedicated wireless link allows clinicians to monitor these devices and program the user-dependent parameters, such as selection of the control sequences and grip thresholds. These SoA devices are enabling complex functionalities, including proportional grasps and configurable gestures, but their control strategies are still based on non-intuitive codification of gestures in muscle contractions.

In this scenario, the deployment of a natural control strategy is a key element; pattern recognition and machine learning techniques are capable to restore a more intuitive control of the artificial hand [35]. Gesture recognition control is based on the assumption that the muscular activation patterns are repeatable among different executions of the same hand gesture. Hence, through the pattern recognition of EMG signals, we can classify hand gestures and grips. The scientific literature proposes a wide analysis of the best sensor configurations [36], classification algorithms [37,38] and actuation controls [39]. Supervised machine learning algorithms, such as Hidden Markov Models (HMM) [40], Linear Discriminant Analysis (LDA) [41], Artificial Neural Networks (ANN) [42] and Support Vector Machines (SVM) [43] provide comparable results of up to 90% of accuracy for the classification of 5 to 10 gestures. EMG-based gesture recognition based on deep learning is starting to be investigated and preliminary results do not show a very strong advancement w.r.t. the SoA [44]. Furthermore, the related classification algorithms are computationally demanding and not suitable for a real-time embedded implementation [45].

One of the main issues of the pattern recognition approach is caused by the variability of the EMG signal during the arm movements. Indeed, differences in the activation patterns caused by the variability of the arm position can cause significant losses in the classification accuracy of the EMG signals. To cope with this, several solutions are proposed in literature [46,47], based on sensor fusion or algorithm tuning. Detailed comparison of acquisition setups and classification algorithms is widely available in literature [48] and it is out of the scope of this work. We focus on the use of the SVM algorithm, which provides an optimal accuracy Vs complexity trade-off and it is suitable for real-time embedded implementation and tight integration with the hand control. Moreover, in our previous work we demonstrated the robustness of the SVM approach against variation of arm postures and electrodes number and positioning [17,49].

Recently, an alternative research solution based on the use of *soft synergies* has been proposed [12,50]. This solution develops the design of a robotic hand with only opening and closing capabilities, but that adapts its grasp on the object being manipulated. Its primary goal is to restore a correct proportional control of the grasping force [51], assuming that the round grip is the most used movement to grasp objects. The hand realized by [52] further refines this approach with the principle of *adaptive synergies* and features 19 joints controlled by a single actuator. This approach focuses on the versatility of the mechanical design of the hand rather than on the EMG-based contraction codification or the gesture recognition It uses the EMG signal for the detection of the hand activation and as a proportional control of the grasping force. This solution is robust and powerful even though the hand can only open and close its adaptive grasp and the finger movements are not independent.

Another interesting approach compares linear and non linear regression methods for proportional and simultaneous control of multiple DoF of the prosthesis [53,54]. The aim of these works is to propose a method to overcome the limitations of a controller that manages only one DoF at time. The regression approach differs from the classification mainly because it provides a continuous output instead of a discrete output associated to a recognized gesture. Nevertheless, the presented setup is based on a large array of sensors and the experiments included only flexion, extension and rotation of the wrist.

There is a lack of complete devices for embedded gesture recognition and control of prosthetics, which is due to the difficulties in performing system-level development and addressing problems ranging from signal acquisition and processing, embedded system design, applied prosthetics and machine learning. The work described in this paper intends to bridge this gap presenting the development and implementation of an intuitive embedded prosthesis controller, as the result of the collaboration between institutes with multidisciplinary competences. We propose a wearable BSN approach for the implementation of an effective and versatile hand controller: an embedded node is dedicated to real-time sensing, processing and actuation, while a portable personal gateway (e.g., smartphone or tablet) provides advanced functionalities such as training and personalization of the recognition algorithms. Our work analyzes the EMG signal acquisition and integrates the use of SVM-based gesture recognition with a real-time controller to provide accurate and robust actuation of a poliarticulated prosthetic hand. The proposed approach is tailored for the implementation on an embedded microcontroller (MCU) and it is evaluated not only validating the recognition algorithm, but also on its end-to-end and timely gesture actuation capabilities.

## 3. Materials and Methods

**Figure 1:**
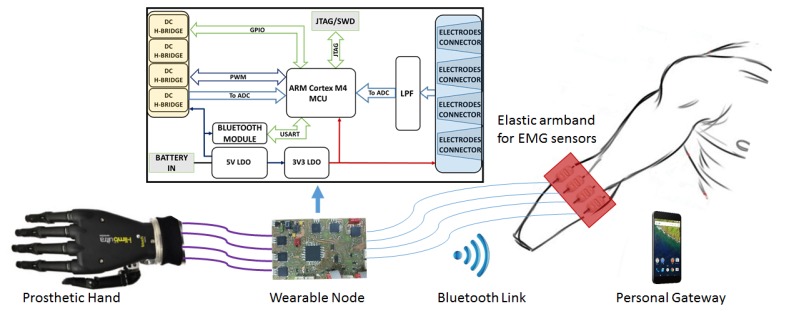
Diagram of the system architecture.

Figure 1 presents the architecture of the proposed system, which is composed by: (1) an elastic armband with 4 EMG sensors; (2) a wearable sensing node responsible for data acquisition and classification, prosthesis actuation and Bluetooth communication; (3) a poliarticulated prosthetic hand and (4) a personal gateway for data acquisition, recognition algorithm training and customization of system parameters.

This heterogeneous BSN architecture aims to maximize the energy efficiency of the node. The signal acquisition, the pattern recognition algorithm and the closed loop control of the hand are executed in real-time on the wearable node, while the algorithm tuning and the SVM training, which do not require real time execution are provided by the personal gateway. Offline studies of EMG gesture recognition typically report recognition accuracy on all collected samples, regardless of their collocation during a gesture. When actuating a prosthesis, only one output per executed gesture is needed and the gesture decision should be made as soon as possible at the start of its execution, while the classification of subsequent samples becomes unnecessary. However, the transient phase at the onset of a gesture is more difficult to classify when compared to a stable on-going contraction. Hence, a robust implementation of a gesture controller has to cope with the initial uncertainty in the recognition and to provide a timely decision for a correct actuation of the hand.

The proposed solution integrates sample-level SVM classification with a high-level Finite State Machine (FSM) to produce accurate and robust control of the prosthesis. For mechanical constraints, the prosthetic hands start every gesture from a reset position, i.e., open hand. Hence, we included this transition to be performed between each executed gesture and used it to improve the robustness and usability of the system. Moreover, during the onset of a gesture, the output of the sample-level recognition is analyzed with a majority voting approach to limit the errors due to the signal transitions and to converge to a decision within a specified time window.

### 3.1. EMG Signal Acquisition

The EMG signal measures the electrical activation of the muscular fibers, generated by the depolarizations of muscular cell membranes named Action Potentials (APs). APs are the result of the propagation of neural impulses from the brain towards the target muscle cells, as a muscular contraction occurs [55].

Surface EMG sensors are composed by two conductive plates connected to the inputs of a differential amplifier, thus sensing the aggregated APs of the underlying muscles. The bandwidth of the EMG signals stays within 2 kHz, while the signal amplitude is contained in the ±10 mV range depending on the diameter of the muscles and on the distance from the sensing elements. In prosthetic applications, EMG acquisition is performed with active analog sensors, to maximize the signal quality reducing noise caused by motion artifacts, fibers crosstalk and floating ground of the human body.

Active EMG sensors perform an analog conditioning of the signal using bandpass filtering and an Instrumentation Amplifier (IA) with a high gain stage. In our implementation, we use the Ottobock 13E200, a family of pre-amplified sensors with single ended output. In such sensors, the EMG signal is filtered, amplified and integrated to reach an output span of 0–3.3 V, ideal for acquisition with the single ended stage ADC integrated in an embedded MCU. Furthermore, the active sensors extract the envelope of the raw EMG signal exploiting the HW circuitry and for this reason, in our application no further feature extraction of the EMG signal is required.

### 3.2. Wearable Controller Node

The main component of the proposed setup is a custom wearable controller board, whose block diagram is illustrated in Figure 1 (top). It is designed on a 4-layer PCB and includes a 32 bit MCU, circuitry for EMG signal acquisition, for the actuation and control of the hand prosthesis and a Bluetooth transceiver for the communication with a host device. The board tolerates a single-voltage power supply from 5.5 to 8 V, to easily fit on commercial prosthetic systems that use standard battery cells varying from 7.2 to 8 V.

The on-board linear regulators provide stable output voltages of 3.3 V and 5 V employed for the different sub-systems. The system is based on an ARM Cortex M4 MCU from NXP. The presence of two independent 16-bit SAR ADCs (*ADC0* and *ADC1*) and of a dedicated PWM output peripheral allow the control of the proposed prosthetic hand minimizing the need for external components and hence the board complexity. Data are acquired at a frequency 500 Hz, which has been shown to be sufficient for gesture recognition applications [49]. On each channel, an RC low pass filters minimizes the high frequency electrical noise. A further resistive voltage divider protects the ADC’s inputs, limiting the signal span to the 0–3.3 V range, while the output span of the Ottobock sensor is 0–5 V.

**Figure 2:**
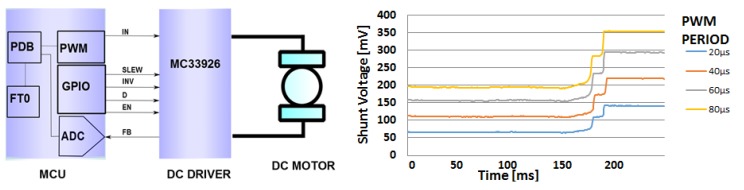
Block diagram of the interface between the MCU and the DC motor driver (**left**) and example motor current absorption curves (**right**).

The DC motors powering the finger actuation of the artificial hand are controlled by an integrated H-bridge driver (MC33926 by NXP), connected with the microcontroller, as described in Figure 2 (left). The FB signal, which goes from the H-bridge to the MCU, returns a feedback on the current absorption of the DC motor, providing to the ADC a voltage signal that is proportional to the current being drawn.

A finger movement is completed when it is completely open, completely closed or if it is halted in grasping an object. In such situations, the DC motor increases its current consumption measured through a shunt resistor and threshold trigger is used to stop the motor. A typical curve of the voltage provided by the H-bridge is reported in Figure 2 (right) for different values of the PWM period.

The hardware configuration is completed with a Bluetooth (BT) transceiver (SPBT2632C2A by STM), allowing the system to communicate with other devices on the body or in its vicinity. The BT standard was chosen for its easy use, optimal throughput and power consumption tradeoff [56] and its versatility to enable communication with a variety of host devices (body gateways, mobiles phones, tablets or PCs). This bi-directional wireless interface is used to stream the acquired EMG data to the PC or to store on the device customized parameters and settings. Data streaming to a host device is employed in order to test the system and to acquire instances of gestures for offline analysis and for the training of the classification algorithm. The trained recognition models and further system settings are then sent back to the embedded device and stored in the MCU’s Flash memory.

### 3.3. Classification Algorithm

The SVM is a supervised learning algorithm that improves the performance of logistic regression methods through the application of the large margin classification [57]. The offline training phase of the algorithm uses labeled instances of data to calculate the optimal separation hyperplane (*maximum margin* hyperplane) between two classes of data through the solution of a convex optimization problem. Such separation plane is represented by a set of data vectors, named the Support Vectors (SVs), which belong to the borders of the two classes and they are used to classify new data instances.

When the two classes are not linearly separable, the input data space can be mapped to a higher-dimensional space through a kernel function to allow an effective separation [59]. Having two classes, denoted as $Cl_{1}$ and $Cl_{2}$, the formula of the decision function to classify a new input instance is:

(1) $$f\left(x\right)=\sum_{i=1}^{N_{SV}}y_{i}\alpha_{i}K\langle x,s_{i}\rangle -\rho \;\;\;\;\;\{\begin{array}{c} f\left(x\right)>0,x\in Cl_{1}\\ f\left(x\right)<0,x\in Cl_{2} \end{array}$$

where $x\in R^{N_{F}}$ is the vector if input features, $s_{i}\in R^{N_{F}}$, $i=1,...,N_{SV}$ are the support vectors, $\alpha_{i}$ are the support values, with $y_{i}$ denoting the class they reference ($y_{i}=+1$ for $Cl_{1}$, $y_{i}=-1$ for $Cl_{2}$) and K〈·,·〉 denotes the kernel function. In our application, the input for the SVM classifier is the 4-dimensional vector of the EMG signals acquired by the electrodes ($N_{F}=4$) and we used the Radial Basis Function (RBF) kernel.

**Figure 3:**
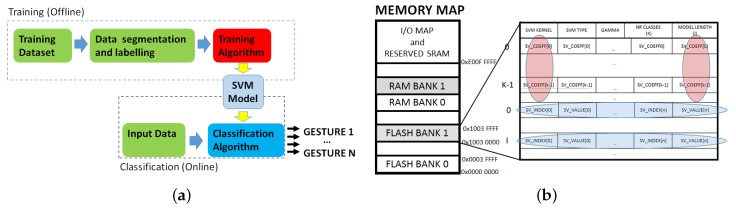
SVM algorithm block diagram (**a**) and memory allocation (**b**).

Since the training of a SVM classifier is computationally demanding for a low power microcontroller and it should be performed only at the setup of the recognition algorithm, it is possible to perform it offline on a PC. This allows the use of a graphical interface to visualize the training data and perform an accurate segmentation without imposing particular limitations on the system architecture. The calculated models are then stored on the MCU where the classification algorithm is executed for a real time recognition of the performed gestures. A diagram of the SVM training and recognition phases is illustrated in Figure 3a.

The libSVM [60] is a popular open source multiplatform library implementing the SVM algorithm, which includes the training and classification functions [60]. The library implementation is adapted for the embedded platform, where the dynamic allocation of the memory structures is not recommended in the design of medical systems [61].

Taking advantage of the pointer manipulation and arithmetic functions offered in C, we stored the SVs in a compact memory area to better exploit the structure of the libSVM’s prediction function, based on nested *for* cycles iterating through the SVs. Figure 3b shows the MCU’s memory structure and the static allocation of the stored parameters.

The pseudocode of the execution of the SVM decision function for the classification of new input data is described in Algorithm 1. The first step is the computation of the RBF kernel mapping of the input vector and the SVs, where the products between the class labels and the support values ($C_{i}=\alpha_{i}y_{i}$) are pre-calculated and stored in the flash memory. These pre-computed coefficients allow to execute the complete decision function between two classes just with 2 nested *for* loops. Finally, the predicted label is the one that has totalized the highest number of binary classifications within all the combination of classes.

In our body-area embedded implementation, the model parameters and the SV values and coefficients are stored maintaining the dependencies between the relative vector positions to access them in an efficient way. The vectors are correctly addressed exploiting the pointer algebra to substitute the allocation of a dynamic list of structures with the indexing of the Flash sector where the SVs and coefficients are stored. The memory requirements of the algorithm include the contribution of a fixed header of 20 Bytes containing the algorithm’s type and configuration parameters and a variable component depending on the number of SVs, data features and recognized classes. Such a variable component has to be multiplied by the size of the used data type to compute the final memory footprint (e.g., 4 Bytes for single precision float).

**Algorithm 1** Multiclass SVM implementation1:$C_{i}=y_{i}\alpha_{i}$2:$v\left[N_{Cl}\right]=\left\{0,0,...,0\right\}$3:**function** Class = *svmpredict*(x)4:**for** i = 1 to $N_{S}$ **do**5:    $K\langle x,s_{i}\rangle =exp\left(-\frac{||x-s_{i}{\left|\right|}_{2}}{2\sigma^{2}}\right)$6:**end for**7:**for** j = 1 to $N_{Cl}$ **do**8:    **for** k = j+1 to $N_{Cl}$ **do**9:        $f\left(x\right)=\sum_{i=1}^{N_{S}}C_{i(jk)}K\langle x,s_{i}\rangle -\rho$10:        **if** f(x)>0 **then**11:           v(j)++12:        **else**13:           v(k)++14:    **end for**15:**end for**16:Class ← index of max(v)

### 3.4. Gateway Application

The proposed system is completed with a mobile personal gateway, which is implemented on a smartphone. The personal gateway application, written in Java on Android, connects via BT to the wearable node and allows to perform a set of test and tuning operations. In particular, the embedded system implements three operation modes: Streaming Mode (SM), Update Mode (UM) and Classification Mode (CM). The transmission of a dedicated control string from the app to the board manages the transitions between the operation modes and the CM is the default mode used for stand-alone control of the prosthesis. SM is used to stream EMG data to the gateway device, useful to test the positioning and functionality of the system and to collect example gestures for a personalized training of the SVM algorithm. Finally, UM allows to update the trained SVM model on the device.

The personalized setup of the system is performed under the supervision of a clinician and it requires the user to correctly position the sensors, test their activation and to collect a training dataset composed by a few repetition of each gesture to recognize. When the SVM model is computed, the app sets the system in UM and manages the model transfer to the MCU. This operation allows to correctly store the personalized model in the Flash memory of the MCU and use it for real-time classification. The communication protocol sends 3 types of packets containing the following:

1. the general configuration values: SVM type, gamma parameter, the number of classes and the number of features.
2. the model parameters: the ρ parameter, the total number of SVs, and the SVs for each class.
3. the SVs and their coefficients, sending one packet for each SV.

**Figure 4:**
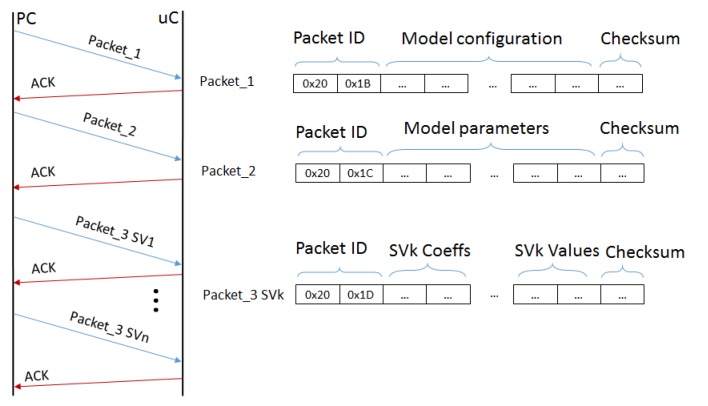
Diagram of the communication between the personal gateway and the wearable node.

When a packet has been sent, the interface waits the Acknowledgment (ACK) message sent from the board before sending a new packet. Each packet has its checksum to check its integrity. Figure 4 shows the transmission scheme between the gateway app and the wearable node.

During the prosthesis control, the classification algorithm and the prosthetic controller run in real time on the embedded board, hence the interaction with the gateway is not required during continuous use.

### 3.5. Control Strategy

For mechanical reasons, prosthetic hands execute the various grasps and movements starting always from a reset state, normally the open hand position. After the execution of a gesture, it is, therefore, necessary to return to the reset configuration before performing another movement. This strategy allows to control the prosthesis movement using only the motor current feedback provided by the integrated driver, since the system always starts from a known configuration.

**Figure 5:**
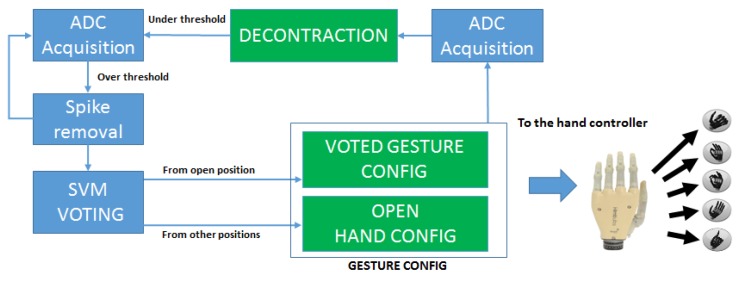
Diagram of the FSM for classification mode.

The diagram of the FSM that controls the the system in CM is represented in Figure 5. In the *ADC Acquisition*, ADC peripheral extracts the mean value of 16 consecutive samples of the 4 EMG channels. The total conversion time of the peripheral is 32 μs for the 16 consecutive samples and this does not affect the real time requirements of the system. The *Spike Removal* block acts as a time trigger to avoid the activation on spurious spikes due to movement artifacts or external noise. The contraction spikes lasting over an activation threshold for less than 100 ms are filtered because they are not related to voluntary hand movements [58]. If the signal lies over the threshold for longer, the system starts the classification of the incoming samples using the on-board SVM models.

Since the onset of a gesture contains the transient phase from the rest to the intended contraction, the recognition of the performed gesture is more difficult at the beginning and gets more reliable as the contraction reaches a steady state. However, a timely response of the prosthesis requires to start the actuation and execute the intended movement as soon as the contraction has started and a natural interaction with the device requires response times below 300 ms [17]. To reach a correct classification within a limited time, in the *SVM Voting* block our system applies the majority voting on 20 consecutive SVM classifications to select the most likely gesture being performed. The gesture with the highest number of classification instances is hence detected as the intended one and the motors are actuated.

The hand controller sets up the finger movement parameters according to the intended gesture. For instance, in the power grasp all the motors receive the command to rotate closing the fingers, while in the precision grasp only index and thumb fingers receive the closing command. The MCU stops the motors exploiting the H-bridge current feedback, which indicates when a finger has reached the correct position. After any performed gesture, the device will need to go back to the reset position (i.e., the open hand) before executing further movements. These transitions are managed by the *Gesture Config* Block, in accordance with the current state of the prosthetic hand. Once a gesture is decoded and actuated, the EMG contraction level is acquired again, waiting for a muscular decontraction, that retriggers the FSM controller. This sequence returns a natural control strategy allowing to keep trace of the current hand position without absolute encoders or other positioning sensors and provides robust control of the prosthesis with an intuitive control strategy.

**Figure 6:**
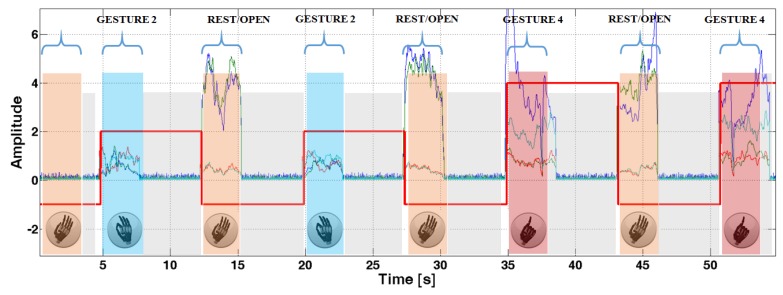
Hand control strategy as executed during a gesture sequence.

Figure 6 shows a sequence of gestures with the system outputs to better explain the proposed strategy. The red bold line shows the output of the controller, where the positive values encode the gestures and the value −1 is reserved for the reset (open hand) position. The control strategy can be summarized as follows. The user performs a gesture to be executed and thanks to real-time recognition with majority voting, responsive but accurate actuation of the hand is provided. Once the prosthesis actuated the gesture, the user can relax the contracted muscles, the decontraction retriggers the classifier and the device will hold this configuration until an open hand gesture is detected. This strategy increases the usability of the controller since the user is not required to hold on the muscular contraction to maintain the grasp, decreasing significantly the level of fatigue.

Furthermore, since the reset hand movement is the only allowed operation to retrigger the prosthesis and prepare it for a subsequent gesture, it is not strictly necessary to classify it, because the FSM does not allow other possible positions reachable after a gesture has been performed. Hence, it is possible to exclude the open hand gesture from the training set and simply detect any muscle contraction to exit the current gesture and reach the reset position. We evaluated the approach both including and leaving out the open hand gesture from the training dataset and we compared the results in terms of performance and efficiency, as presented in the following section.

## 4. Experimental Results

With our experiments, we demonstrate that using a proper control strategy on top of the classification algorithm improves greatly the accuracy and the robustness of the final gesture recognition. In prosthetics, a fair comparison between different approaches is not trivial, because differences on the setup have a great impact of the performance. Furthermore, when considering real-time control of a prosthesis, the sample recognition accuracy does not evaluate the complete system, which is better evaluated by its ability to timely perform the intended user movement, with robustness to spikes, false contractions and misclassifications. We can call this metric the *end-to-end* success ratio. To cope with this issues, and compare our solution with a literature benchmark we initially validated our system offline on an EMG dataset, the NINAPRO database, a recently released extensive collection of hand gestures for EMG recognition. The NINAPRO database collects up to 52 different hand gestures from 27 subjects, recorded with an accurate setup using a hand-tracking glove and 10 Ottobock EMG sensors on the forearm. From such dataset, we selected the same gestures as the ones used in our application and we constructed an interleaved dataset inserting open gestures between the others, to have a fair comparison also of the FSM controller. Regarding the number of sensors, we used the 10 EMG channels as the input vectors ($N_{F}=10$). For the performance evaluation on benchmark datasets, we replicated the functionality of the recognition algorithm and of the controller in a simulated environment using Matlab. Hence, we tested the system with the data from the NINAPRO database, evaluating the number of correctly executed gestures.

The end-to-end classification accuracy is evaluated over 10 repetitions of sequences composed by a gesture followed by an open hand gesture. Each user performed 10 repetitions of 3 gestures: power grasp, pointing index and precision grasp. We collected a total of 60 consecutive movements from each user. On this sequence, we evaluated the number of wrong gestures as executed by the proposed control strategy. To have a comparison with the classical classification accuracy, an error of 1 in 10 gestures is equivalent to an error rate of 10% (i.e., 90% accuracy), while an error of 1 in 60 gestures corresponds to an error rate of 1.66% (98.4% accuracy).

**Figure 7:**
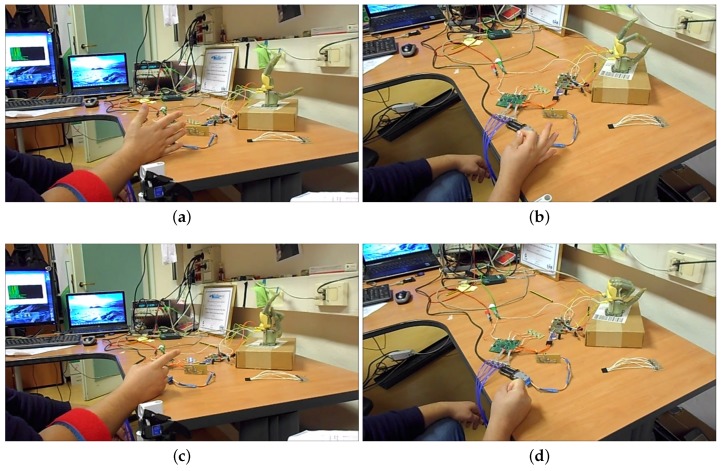
Hand controller during the real time experiment with healthy subjects in open hand (**a**); precision grasp (**b**); point index (**c**) and power grip (**d**). In figures (**a**,**c**) it is possible to see the armband with the EMG sensors.

Then we tested the system in real time on 5 healthy subjects, in our laboratory and on 4 transradial amputees with 1/3 distal amputation, at the INAIL center. All participants provided informed written consent to take part in the experiment, which was approved by the Ethical Committee of the University of Bologna (Italy). The muscular contractions during the gesture execution is 3 s long and between each contraction there are 3 s of rest. On the healthy subjects, the 4 EMG sensors were placed in correspondence of the *flexor radialis carpi, flexor ulnaris carpi, extensor communis digitorum and extensor carpi ulnaris.* These muscles were targeted as the actuators of the movements analyzed in the experiment, accordingly to [17]. Figure 7 shows the real time experiment on the healthy subject with the I-Limb hand. Regarding the amputee subjects, since the muscular structure of the forearm is compromised, the sensors were placed at equal distance between each other, starting from the position of a working or partially working muscle, found with tactile analysis.

Since the feature extraction is performed in the analog domain directly on the Ottobock sensors, each sample that comes out from the ADC can be classified by the SVM. To increase the robustness of the system, the majority voting is applied over 20 consecutive samples, allowing a classification output every 40 ms. Results reported in Table 1 show the end-to-end execution accuracy and the number of SVs in the trained models on NINAPRO (offline) and on healthy and amputee subjects (online).

The first column (*Accuracy*) shows the cross-validation recognition accuracy calculated on all the samples of the training dataset, which confirms that the classifier performance is aligned with the SoA. Under the columns labeled *COMPLETE*, we show the number of SVs and the end-to-end error rates using a SVM model that includes the open hand gesture to be recognized. In the *REDUCED* case, we excluded the open hand gesture and simply assume such gesture is performed when a contraction is detected after any other gesture.

The evaluation of the proposed control strategy shows similar values of accuracy and of end-to-end classification rates for the *COMPLETE* and *REDUCED* approaches. Our solution with only 4 EMG sensors shows comparable results to the one obtained with the NINAPRO dataset, where EMG acquisition is based on an array of 10 sensors, hence more complex and expensive.

**Figure 8:**
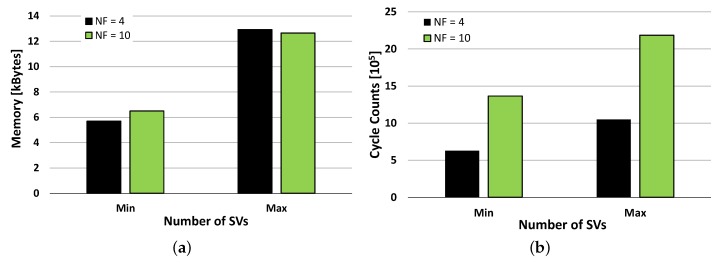
Memory footprint of the SVM models in kBytes (**a**), computation requirements in clock cycles (**b**), varying the number of SVM features.

Maintaining a low complexity is a key point in the development of an efficient embedded system and its design and implementation should be tailored to optimally employ the hardware resources. The number of EMG channels, and hence the dimensionality of the input data vectors, beside the impact on the system cost has a major impact on the amount of the required memory and on the computational load of the recognition algorithm. The proposed SVM is implemented using single precision floating point representation for the SVs, their coefficients and the input signals. This directly impacts on the hardware resources needed for model storage and real-time execution, which we evaluated on the used MCU. The minimum and maximum memory requirements given by the trained models for the two analyzed setups (our and NINAPRO healthy subjects) are reported in Figure 8a. The two solutions have very similar memory occupation and this is given by their combinations of input features and the resulting SVs. Our solution has a smaller number of input features ($N_{F}=4$) and results in models with a higher number of SVs ($N_{S}=178÷404$). In contrast, the NINAPRO setup has a higher number of input channels ($N_{F}=10$) and results in models with a lower number of SVs ($N_{S}=116÷226$). Overall the two have similar memory occupation of up to 12 kB, which can be handled by the proposed MCU equipped with 64 kB of Flash.

The run-time computational load of the application is composed by the signal acquisition, the SVM prediction and the control FSM. The SVM prediction dominates the workload and sets the requirements for real-time execution. Figure 8b shows its execution time, expressed in CPU cycle counts, as measured using the MCU’s internal cycle counter (CYCCNT) register. We analyzed the two setups, having input vectors of 4 and 10 and we again measured the performance for the best and worst cases (min and max number of SVs). The computational times are again influenced by the number of input features, the number of recognized gestures and the resulting number of SVs, but this time the obtained combinations do not result in similar requirements. In fact, the proposed approach outperforms the NINAPRO setup and leads to a reduced computation time in all combinations.

**Figure 9:**
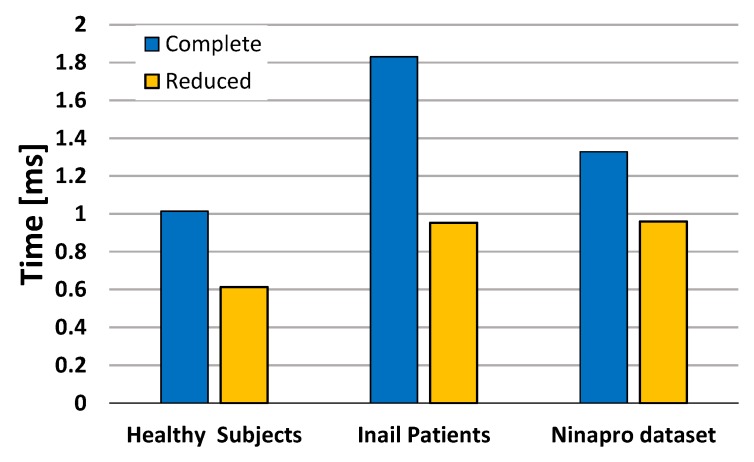
Computation times expressed in ms for the two analyzed setups.

Figure 9 shows the quantitative differences of the computational load of the algorithm with healthy subjects, amputees and with NINAPRO data, considering the complete and reduced datasets. To compare the complete and reduced approaches, we consider the mean execution times of the 3 classes of subjects on the proposed MCU. The SVM reaches the best performance with the healthy subjects, because the activation patterns of the different gestures are well separated due to the good condition of the forearm muscles. With the NINAPRO healthy subjects the SVM reaches comparable performance, even though the execution is slightly slower.

The test on amputee patients demonstrates that the computation of the classifier’s output requires a significant increase of time with the complete dataset, due to the larger SVM model. In fact, the compromised residual muscles of the amputees produce more confused activation patterns and the SVM algorithm needs more SVs to define the separation boundary between the classes. Nevertheless, with the proposed control strategy, it is possible to reduce the number of SVs of the model by the elimination of the open hand gesture. In this case, we obtain a significant reduction of the number of SVs and consequently of the computation time that becomes comparable to the performance obtained on healthy subjects with a redundant sensor setup. The experimental evaluation shows that the proposed hardware and software implementation of this BSN is tailored to meet the requirements of a real time wearable controller for a prosthetic hand through the multimodal approach of the desing, from system architecture to algorithm and control level.

## 5. Conclusions

Recent research efforts explore different solutions for a natural control of poliarticulated hand prostheses, employing advanced gesture recognition techniques or combining myoelectric proportional control with innovative mechanical design of the hand based on adaptive synergies. However, the design of a complete system must draw on knowledge from various research fields and nowadays a considerable gap persists between research and lab evaluation of such solutions and their real-life usability and implementation into successful commercial products.

The contribution of the paper is in the complete design and the final deployment of a body area controller for a poliarticulated prosthetic hand, based on natural control strategy. The work described in detail the hardware and software implementation of an efficient embedded pattern recognition algorithm and of a reliable control strategy of the poliarticulated hand. We validated our approach by applying it to data from the NINAPRO database that represents the largest and most complete dataset for EMG gesture recognition analysis, obtaining again minimal errors.

Moreover, we presented the online performance of our wearable node in terms of end-to-end recognition ratio, evaluating the accuracy and timing of the system to recognize and execute complete hand gestures, as performed by healthy and amputees subjects. The proposed approach resulted having an average error rate of 1 misclassified gesture every 60, for both healthy and amputee subjects. The innovative complete control strategy has been implemented on the embedded system, which allows its real-time execution. To ensure a robust but timely response of the system, we applied a majority voting approach to the output of the SVM classifier, obtaining a recognition time of 36 ms, in the worst case. Future works will target the consolidation of an advanced prototype to enable a detailed evaluation of usability in real life scenarios and the comparison with SoA solutions.

## Figures and Tables

**Table 1:** Gesture recognition accuracy and errors in hand movements decoding of the proposed controller.

**Healthy Subjects**	**COMPLETE**			**REDUCED**	
	**Accuracy**	**SVs**	**FSM Errors**	**SVs**	**FSM Errors**
S1	88.01	178	0	124	0
S2	89.76	312	0	209	0
S3	89.21	229	0	155	0
S4	86.34	404	0	204	0
S5	83.49	311	0	206	0
MEAN	87.37	296	0	179	0
**INAIL patients**	**COMPLETE**			**REDUCED**	
	**Accuracy**	**SVs**	**FSM errors**	**SVs**	**FSM errors**
S1	94.86	166	0	55	0
S2	93.65	262	0	38	0
S3	81.38	393	1	290	0
S4	86.43	1316	1	729	1
MEAN	89.09	534	<1	278	<1
**NINAPRO data**	**COMPLETE**			**REDUCED**	
	**Accuracy**	**SVs**	**FSM errors**	**SVs**	**FSM errors**
S1	90.46	142	0	116	0
S2	93.49	116	4	107	4
S3	92.89	132	0	67	0
S4	92.15	226	0	169	0
S5	90.15	158	0	114	0
MEAN	91.83	155	<1	112	<1
